# Comparison of Different Antiviral Regimens in the Treatment of Patients with Severe COVID-19: A Retrospective Cohort

**DOI:** 10.3390/medicina59020260

**Published:** 2023-01-29

**Authors:** Mohammad E. M. Mahfouz, Afrah A. Alharthi, Nada M. Alsalmi, Ahad A. Alnemari, Amjad A. Alwagdani, Reem K. Alghamdi, Razan A. Almakki, Mubarak R. Al Yami, Ahmed N. Alghamdi, Afaf S. Osman, Ahmed S. Abdel-Moneim, Dalia Y. Kadry

**Affiliations:** 1 Department of Surgery, College of Medicine, Taif University, Taif 21944, Saudi Arabia; 2 College of Medicine (Graduate Students), Taif University, Taif 21944, Saudi Arabia; 3 King Faisal Medical Center (KFMC) Taif 26514, Saudi Arabia; 4 Department of Microbiology, College of Medicine, Taif University, Taif 21944, Saudi Arabia; 5 Medical Pharmacology Department, Faculty of Medicine, Cairo University, Cairo 11562, Egypt; 6 Department of Microbiology, National Cancer Institute, Cairo University, Cairo 11796, Egypt

**Keywords:** antiviral, clinical outcome, coronavirus, disease severity, COVID-19, SARS-CoV-2, Saudi Arabia

## Abstract

The severe acute respiratory syndrome coronavirus 2 (SARS-CoV-2) causes respiratory disorders, with disease severity ranging from asymptomatic to critical manifestations. The current retrospective study compared the efficacies of different antiviral regimens used in patients suffering from severe COVID-19 disease from 19 January 2020 to December 2021 in a single center in Saudi Arabia. In total, 188 patients were enrolled in the current study, including 158 patients treated with different antiviral regimens, and 30 who did not receive any antiviral treatment. Different antiviral regimens, including favipiravir, remdesivir, oseltamivir, favipiravir/remdesivir, and favipiravir/oseltamivir were adopted. The effects of using different antivirals and antibiotics on the survival rate were evaluated, as well as the presence of comorbidities. Among all severely affected patients, 39/188 (20.7%) survived. Both age and comorbidities, including diabetes and hypertension, were significantly correlated with high case fatality following SARS-CoV-2 infection. Remdesivir alone and the combination of favipiravir and remdesivir increased the survival rate. Surprisingly, both imipenem and linezolid helped in the deterioration of disease outcome in the patients. A negative correlation was detected between increased mortality and the use of favipiravir and the use of either imipenem or linezolid. Among the compared antiviral regimens used in the treatment of severe COVID-19, remdesivir was found to be an effective antiviral that reduces COVID-19 case fatality. Antibiotic treatment using imipenem and/or linezolid should be carefully re-evaluated.

## 1. Introduction

Coronavirus disease 2019 (COVID-19) is caused by severe acute respiratory syndrome coronavirus 2 (SARS-CoV-2), which is a virus related to the subgenus *Sarbecovirus* and genus *Betacoronavirus* within the family *Coronaviridae* [1]. Globally, COVID-19 has been confirmed in more than 661 million cases, with 6,700,519 fatal cases reported to WHO as of 13 January 2023 [2]. The virus is highly transmissible among humans through both direct and indirect contacts [3].

SARS-CoV-2 has an incubation period of 5–7 days; however, it can take up to 14 days to develop symptoms after being exposed to the virus [4]. COVID-19 can be asymptomatic or symptomatic. In symptomatic cases, the disease severity can be mild, moderate, severe, or critical [5]. Patients who suffer from the severe form of the disease develop a hyperinflammatory state that could lead to a critical condition. The asymptotic cases are characterized by respiratory failure, acute respiratory distress syndrome, septic shock, thromboembolism, and/or multi-organ failure [6]. Acute kidney and cardiac injuries are among the most common impacts [7,8].

Older age, smoking, and underlying diseases such as diabetes, hypertension, cardiac diseases, chronic lung diseases, and cancer have all been identified as risk factors for the development of severe diseases and fatal consequences [9].

Antiviral therapy is used effectively in the treatment of several viral infections. Antiviral drugs help in easing symptoms and shortening the duration of the illness. On 22 October 2020, the FDA approved Veklury (remdesivir) for use in adults and pediatric patients (above 12 years of age). Remdesivir (GS-5734) inhibits the viral RNA-dependent RNA polymerase (RdRp) with in vitro inhibitory activity. It was found to be active against both the severe acute respiratory syndrome coronavirus 1 (SARS-CoV-1) and the Middle East respiratory syndrome (MERS-CoV). Owing to its capability to inhibit SARS-CoV-2 in vitro, it was discovered early as a promising therapeutic candidate for COVID-19 [10].

Lopinavir/ritonavir and favipiravir are antivirals that have been used in the treatment of COVID-19 and are currently being employed in different clinical trials, as previously reviewed [11]. Favipiravir triphosphate is a purine nucleoside analog that inhibits RdRp in a competitive manner. It is effective against influenza viruses, RNA viruses associated with viral hemorrhagic fever, and SARS-CoV-2 [12]. Oseltamivir is a neuraminidase inhibitor that has been successfully used as an antiviral treatment against influenza A and B viruses. Although it is used as a therapeutic option in some studies, its efficiency in COVID-19 treatment remains controversial [13,14].

Repurposed drugs, including chloroquine (CQ), hydroxychloroquine (HCQ), and ivermectin, have also been used in COVID-19 treatment [11]. CQ and HCQ aminoquinolines have been used for treating malaria and chronic inflammatory disorders, such as systemic lupus erythematosus and rheumatoid arthritis. Their efficacy in treating patients with COVID-19 infection is attributable to their antiviral and anti-inflammatory activities [15,16]. Ivermectin is an anthelminthic drug that can bind to the SARS-CoV-2 S protein, human ACE-2, and TMPRSS2 receptors and inhibit virus entry to host cells [17].

Other supportive drugs that are essential in reducing inflammatory responses, including corticosteroids and anti-IL-6 Mab, have also been used in COVID-19 treatment. Corticosteroids work through their anti-inflammatory and immunosuppressive properties to reduce damage in different tissues. Glucocorticoids inhibit nuclear transcription factor-κB signaling and inflammatory factor transcription and translation. Thus, they are used as anti-inflammatory drugs in different medical conditions, such as bacterial or viral pneumonia. Corticosteroids have also been used in the past during SARS-CoV-1 and MERS-CoV outbreaks. Accordingly, the use of corticosteroids in the recent COVID-19 pandemic is based on the genetic similarities of SARS-CoV-2 with SARS-CoV-1 and MERS-CoV [18].

Antibiotics have been widely used as part of a treatment protocol for COVID-19 in many countries. In addition to the struggles related to antibiotic resistance, most of the guidelines recommend treatment with antibiotics. The WHO recommended that antibiotic therapy or prophylaxis should not be used in patients with mild/moderate COVID-19 unless it is justifiable. Azithromycin is recommended for treating respiratory, urogenital, dermal, and other bacterial infections and exerts immunomodulatory effects in chronic inflammatory disorders [19]. The use of these antibiotics has been associated with clinical improvement and even reversal of cytokine storms in some infections caused by RNA viruses [20].

Limited information is available regarding the efficacy of different antivirals used in reducing both disease severity and mortality rate. Accordingly, we aimed to investigate the various treatment regimens used to treat COVID-19 patients and how they influence clinical outcomes.

## 2. Materials and Methods

### 2.1. Ethical Approval

This research proposal was approved by No. 353 on 9 May 2021, from IRB of the Research and Studies Section of the Directorate of Health Affairs in Taif, Saudi Arabia.

### 2.2. Patients

The current retrospective study was conducted at the King Faisal Medical Complex in Taif city, Saudi Arabia. The exclusion criterion included patients who tested negative for COVID-19 or COVID-19-positive patients with mild (no shortness of breath or normal chest X-ray) to moderate illness (lower respiratory distress or imaging with oxygen saturation ≥94% on room air). The following were the inclusion criterion: laboratory-confirmed COVID-19 patients who suffered from a severe form of the disease and were admitted to the intensive care unit (ICU). Patients with an oxygen saturation <94% in room air, a respiratory rate >30 breaths/min, or lung infiltration of >50% were considered severe COVID-19 patients and were enrolled in the study [21]. In total, 188 patients, including 69 females and 119 males with an age range of 21 to 93 years, were enrolled in the study. Laboratory diagnosis was conducted using real-time reverse transcription polymerase chain reaction or cobas^®^ SARS-CoV-2, which targets the conserved regions within the ORF 1a/b and E genes (Roche, Basel, Switzerland). The test was conducted in the regional reference laboratories belonging to the Saudi Ministry of Health.

### 2.3. Clinical Data

Data were collected retrospectively from 19 January 2020 to 31 December 2021. Demographic data, including age and sex; clinical data such as clinical signs, comorbidities (diabetes, hypertension, cardiac diseases, and cancer), and clinical findings (respiratory rate, chest X-ray findings, and high-resolution computed tomography); and treatment regimens (antiviral, antibiotic, corticosteroids, and anti-IL-6) were collected from the patients’ files.

### 2.4. Treatment Regimens

The ICU patients who tested positive for COVID-19 were grouped into the following clusters: (i) patients who received supportive treatment only (no antiviral, but antibiotic, anti-pyretic, anti-histaminic, and/or cortisone therapy); (ii) patients who received remdesivir (a single IV injection of 200 mg on the first day and then 100 mg once daily for 5 days) beginning from 20 November 2020; (iii) patients who received favipiravir (oral administration of 1800 mg twice daily on the 1st day followed by a twice daily dose of 800 mg for 7 days); (iv) patients who received oseltamivir (oral administration of 75 mg twice daily for 5 days); (v) patients who received both remdesivir and favipiravir with the same dose and treatment duration as for groups ii and iii; and (vi) patients who received both favipiravir and oseltamivir with the same dose and treatment duration as for groups iii and iv.

### 2.5. Statistical Analysis

The results were expressed in numbers and percentages and analyzed by Crosstabs analysis with chi-square and Spearman’s analyses using SPSS version 16. A multivariate analysis of variance was used to screen the benefits of administering individual drugs to reduce the mortality rate among the patients.

## 3. Results

### 3.1. Demographic and Clinical Data

The case fatality was high (149/188, 79.3%), including 91/119 (76.5%) of male patients and 58/69 (84.1%) of female patients (Table 1). All patients suffered from different degrees of lung lesions, including uni- or bilateral infiltration, glass ground consolidation, pleural effusion, and/or bilateral fibrosis.

The overall results revealed no correlation between sex and mortality rate (R = −0.09) (Table 1). The patient age ranged from 21 to 93 years, and there was a significant variation among age groups in relation to the overall mortality rate. In addition, a highly significant correlation was detected between age and mortality rate (R = −0.455). Both diabetes and hypertension constituted risk factors in the fatal cases (*p* < 0.005) (Table 1).

### 3.2. Effect of Different Variables on the Clinical Outcomes of Using Antiviral Regimens in Critically Ill COVID-19 Patients

The use of remdesivir significantly reduced the mortality rate in the treated patients (*p* < 0.001), and there was a high correlation between using remdesivir and increased recovery rate (R = 0.547). In contrast, the use of neither favipiravir nor oseltamivir improved the survival rate. Surprisingly, there was a negative correlation between the use of favipiravir and the mortality rate (R = −0.292) (Table 1). A combination of two antiviral regimens was adopted in some patients. All five patients who were treated with both oseltamivir and favipiravir showed fatal consequences. Twenty-seven patients were treated with both remdesivir and favipiravir. Although not statistically significant, the latter regimen resulted in an improved survival rate (29.6%) (Table 1).

We screened the effect of different variables using crosstabs with different patient groups that were given different antiviral regimens. Remdesivir use was correlated with an enhanced survival rate in both sexes (*p* < 0.001, R = 0.598) (Table 2). Similarly, the combination of remdesivir and favipiravir enhanced the recovery rate in males (*p* < 0.048, R = −0.318) (Table 2). Antiviral use, in general, significantly reduced the fatal consequences in different age groups (*p* = 0.027); however, no significant correlation was detected (R = −0.132) (Table 2). Age was significantly correlated with the overall fatal cases (Table 1). However, there was no statistical variation among the age groups treated with different antiviral regimens and the recovery rate (Table 2). Diabetes (*p* = 0.019, R = 0.185), hypertension (*p* < 0.001, R = 0.329), and cardiac diseases (*p* = 0.033, R = 0.166) were significant risk factors associated with high mortality rates, especially in patients treated with favipiravir (Table 2). The fatal consequences of antiviral use are not aggravated by diabetes. However, it was found to be a risk factor for the increased mortality rate in the favipiravir-treated group (chi-square *p* < 0.001, R = 0.381) and not in other treated groups. Interestingly, the fatal consequences in the remdesivir-treated group were reduced in the diabetic group (*p* < 0.001, R = 0.581). Similarly, hypertension and cardiac diseases were risk factors that increased the fatality rate in the favipiravir-treated group [(*p* < 0.011, R = 0.329) and (*p* = 0.033, R = 0.166) for hypertension and cardiac diseases, respectively]. Accordingly, diabetes, hypertension, and cardiac diseases were risk factors and correlated with the increased mortality rate in the favipiravir-treated group.

### 3.3. Impact of Using Supportive Therapy

Most patients [161 (85.6%)] were treated with dexamethasone, and only 27 (15.4%) patients did not receive dexamethasone; no significant differences were found between the two groups (Table 1). Anti-IL-6 was adopted in only 8 patients, 7 (87.5%) of whom died (Table 1).

### 3.4. Using Antibiotic Regimens

Antibiotics were prescribed in most patients in the current study (185/188). Different antibiotic combinations were used in the COVID-19 patients with secondary bacterial infections. Moxifloxacin was used in most patients (90/188, 47.8%), followed by imipenem (71/188, 37.7%), linezolid (65/188, 34.5%), vancomycin (56/188, 29.7%), and levofloxacin (37/188, 19.6%). Azithromycin was used only in 17 (9%) of the treated patients. Meropenem treatment with favipiravir (*p* < 0.016, R = −0.196), as well as the treatment combining remdesivir and favipiravir (*p* < 0.001, R = 0.423), showed a significant increase in fatal cases. An increased mortality rate was also detected in the group treated with linezolid combined with favipiravir (*p* < 0.015, R = 0.200) and in the group treated with imipenem combined with favipiravir (*p* = 0.001, R = 0.518). A significant increase in mortality rate was also detected in patients treated with a combination of remdesivir, favipiravir, and moxifloxacin (Table 3).

### 3.5. Multivariate Analysis of Variance for Determining Significant Variants

A multivariate analysis of variance revealed that age (*p* > 0.001), diabetes (*p* < 0.005), hypertension (*p* < 0.005), remdesivir (*p* < 0.001), favipiravir (*p* < 0.001), imipenem (*p* < 0.001), and linezolid (*p* < 0.004) significantly affected the mortality rate of patients with severe forms of COVID-19 (Table 4). The younger the age, the lower the morality rate, and the older the age, the higher the mortality rate. COVID-19 patients suffering from the comorbidities of diabetes and hypertension were at a higher risk of increased mortality rate. Remdesivir use was associated with a significant reduction in the mortality rate, while favipiravir use was associated with increased mortality among patients with severe COVID-19. Increased mortalities were detected when treating severe COVID-19 patients with imipenem, meropenem, linezolid, and moxifloxacin (Table 3). However, according to the multivariate analysis results, only imipenem (*p* < 0.001) and linezolid (*p* < 0.004) were associated with an increased mortality rate (Table 4).

## 4. Discussion

To date, four antiviral drugs have been FDA-approved for use in COVID-19 cases: veklury (remdesivir), approved on 22 October 2020; olumiant (baricitinib), approved on 10 May 2022; paxlovid (nirmatrelvir and ritonavir), approved on 22 December 2021; and lagevrio (molnupiravir), approved on 23 December 2021 [22]. Both veklury and olumiant are used in the treatment of severe COVID-19 cases. The former is used for intravenous administration in adults and children with an age of 12 years or higher, and it mainly prevents virus replication by inhibiting SARS-CoV-2 RdRp. Olumiant is an oral pill, which is a repurposed drug that possesses an anti-rheumatoid arthritis effect that reduces inflammation, together with having antiviral activity by preventing virus entry into target cells. In contrast, both paxlovid and lagevrio are used in mild-to-moderate COVID-19 cases. Paxlovid, an oral pill, contains two types of medications: nirmatrelvir (block virus replication) and ritonavir (protease inhibitor). Molnupiravir also inhibits RdRp by acting as a ribonucleoside analog for viral RNA polymerase [22]. Meanwhile, many drugs and potential drugs are available for SARS-CoV-2 treatment. Favipiravir (T-705) is a viral RNA polymerase inhibitor that was approved for marketing in Zhejiang Province, China, on 16 February 2020. Other drugs that were tested for their reactivities to SARS-CoV-2 either in vitro or in vivo included CQ and HCQ, ribavirin, penciclovir, nitazoxanide, and nafamostat [11,23]. Ivermectin, a repurposed drug found to possess antiviral activity against dengue fever [24], was also assumed to possess antiviral potential against SARS-CoV-2 [17,25].

In the current study, we compared different antivirals used in treating patients with severe COVID-19 in a retrospective manner. There was no correlation between the overall case fatality and the sex of the patients. High mortality rates in both sexes were detected in most age groups (41–93 years old), with a highly significant correlation between age and mortality rate. This finding agrees with previous studies in which COVID-19 mortality was found to be strongly dependent on age [26,27,28].

Remdesivir was the first FDA-approved drug for treating COVID-19 patients. In the current study, remdesivir successfully reduced the mortality rate in COVID-19 patients when used alone or in combination with favipiravir. Similarly, a study found at least a 7% reduction in the mortality rate of patients treated with both remdesivir and dexamethasone. The study tested 1694 individuals as a part of a national cohort [29]. In another study, remdesivir treatment resulted in a recovery rate of 74.4% in treated patients versus 59.0% in the non-treated group [30]. In contrast, a study supported by the WHO reported the lack of benefits of remdesivir compared to a placebo in the mortality rate [31].

Favipiravir is an oral drug that was approved by the Chinese FDA for use in clinical trials of COVID-19 patients in early 2020. It showed promising results, especially in patients with mild-to-moderate disease severity [32,33]. Its use reduced the hospitalization time, as well as the probability of deterioration in patients’ diseased conditions by reducing the use of mechanical ventilation [32]. However, our study revealed that favipiravir could not reduce the mortality rate in COVID-19 patients with severe disease conditions. Our results agree with previous studies that confirmed the lack of a significant impact of favipiravir in patients in terms of improving their clinical condition and reducing the requirement of oxygen supplementation [34]. Our results also agree with a previous study that confirmed the lack of a satisfactory effect of favipiravir use on the mortality rate [32]. In the current study, oseltamivir, an antiviral against influenza A and B viruses, was not found to be effective in reducing the mortality rate in COVID-19 patients. Our finding agrees with many studies that confirmed the lack of beneficial effects of using oseltamivir in COVID-19 treatment [14,35,36,37].

Different antibiotic combinations were used in the COVID-19 patients in the current study. The use of azithromycin was not correlated with a reduced mortality rate in COVID-19 patients. Surprisingly, significantly high mortality rates were found when using either imipenem or linezolid. However, in such patients, no significant correlation was detected in their use along with the administration of antiviral drugs. Indeed, antibiotics can save the lives of critical COVID-19 patients; however, we found that fatal consequences in COVID-19 patients were not alleviated using antibiotics unless there was evidence of a secondary bacterial infection [38]. Accordingly, special care should be taken when using antibiotics to avoid the risk of developing resistant bacterial strains.

Furthermore, corticosteroids were used in the current study in most patients and were found to have a beneficial effect in reducing mortality. The WHO and the CDC recommend against the routine use of corticosteroids in patients with COVID-19-related pneumonia unless they are used for treating comorbidities such as asthma [39,40,41].

## 5. Conclusions

The current study confirms the benefit of using remdesivir in increasing the survival rate in severe cases of COVID-19. The finding that the use of certain antibiotics is associated with increased mortality needs further investigation. Although this study confirms the benefit of using remdesivir against COVID-19, it may vary with the cohort, age group, comorbidities, severity score, and initiation of antivirals post-infection.

## 6. Limitations

The patient groups in the current study were heterogeneous in regard to age, which ranged from 21 to 93 years, and sex (119 males and 69 females). The frequency of favipiravir use was higher in comparison to other antiviral drug regimens. There were some confounding results, especially with regard to missing laboratory or clinical information of some patients.

## Figures and Tables

**Table 1:** Significance of age, sex, comorbidities, antiviral use, and supportive treatment on the survival rate of critical COVID-19 patients.

Variable		Non-Fatal Cases (39)	Fatal Cases (149)	Total (188)	Significance *
Sex	Male	28 (23.5%)	91 (76.5%)	119 (63.3%)	*p* < 0.149
	Female	11 (15.9%)	58 (84.1%)	69 (36.7%)	R = −0.09
Age (years)	21–30	4 (100%)	0 (0%)	4 (2.1%)	
	31–40	7 (53.8%)	6 (46.2%)	13 (6.9%)	*p* < 0.001 ** R = −0.455
	41–50	12 (46.2%)	14 (53.8%)	26 (13.8%)	
	51–60	9 (26.5%)	25 (73.5%)	34 (18.1%)	
	61–70	5 (10.9%)	41 (89.1%)	46 (24.5%)	
	71–80	0 (0%)	37 (100%)	37 (19.7%)	
	81–93	2 (7.1%)	26 (92.9%)	28 (14.9%)	
Diabetes	YES	16 (13.7%)	101 (86.3%)	117 (62.2%)	R = −0.202
	NO	23 (32.4%)	48 (67.6%)	71 (37.8%)	*p* < 0.005 **
Hypertension	YES	12 (12.8%)	82 (87.2%)	94 (50.0%)	R = −0.203
	NO	27 (28.7%)	67 (71.3%)	94 (50.0%)	*p* < 0.005 **
Cardiac diseases	YES	8 (16.7%)	40 (83.3%)	48 (25.5%)	R = −0.059
	NO	31 (22.1%)	109 (77.9%)	140 (74.5%)	*p* < 0.258
Cancer	YES	0 (0%)	6 (100%)	6 (3.2%)	R = −0.093
	NO	39 (21.4)	143 (78.6%)	182 (96.8%)	*p* < 0.243
	YES	3 (22.1%)	123 (77.8%)	158 (84%)	*p* < 0.202 R = 0.08
Using antivirals	NO	4 (13.3%)	26 (86.7%)	30 (16%)	
Treatment with a single antiviral	Remdesivir	17 (85%)	3 (15%)	20 (12.7%)	*p* < 0.001 ** R = 0.547
	Favipiravir	9 (9.3%)	88 (90.7%)	97 (61.4%)	*p* < 0.001 ** R = −0.292
	Oseltamivir	1 (11.1%)	8 (88.9%)	9 (5.7%)	*p* < 0.409 R = −0.053
Treatment with two antivirals	Remdesivir and favipiravir	8 (29.6%)	19 (70.4%)	27 (17.1%)	*p* > 0.262 R = 0.055
	Favipiravir and oseltamivir	0 (0.0%)	5 (100%)	5 (3.2%)	
Dexamethasone	YES	32 (19.9%)	129 (80.1%)	161 (85.6%)	R = −0.108
	NO	7 (25.9%)	20 (74.1%)	27 (15.4%)	*p* < 0.110
Anti-IL-6	YES	1 (12.5%)	7 (87.5%)	8 (4.3%)	R = −053
	NO	38 (21.1%)	142 (78.9%)	180 (95.7%)	*p* < 0.409

Statistical analysis was conducted using chi-square and Spearman’s correlations. * Statistical analysis was conducted using chi-square and Spearman correlations. ** Variables showed a highly significant *p* value using chi-square.

**Table 2:** Effects of different variables on the clinical outcomes of using antiviral regimens in critical COVID-19 patients.

Variable		No Antivirals (30)		Use of Antivirals (158)		Remdesivir (20)		Favipiravir (97)		Oseltamivir (9)		Remdesivir and Favipiravir (27)		Favipiravir and Oseltamivir (5)		Non-Fatal Outcome (39)	Fatal Outcome (149)	Total (188)
		N ^a^	F ^b^	N ^a^	F ^b^	N	F	N	F	N	F	N	F	N	F			
Sex	Male	4	17	24	74	7 *	1	8	50	1	6	8 *	14	0	3	28 (23.5%)	91 (76.5%)	119 (63.3%)
	Female	0	9	11	49	10 *	2	1	38	0	2	0	5	0	2	11 (15.9%)	58 (84.1%)	69 (36.7%)
Age (years)	21–30	0	0	4	0	3	0	1	0	0	0	0	0	0	0	4 (100%)	0 (0%)	4 (2.1%)
	31–40	2	0	5	6	3	0	0	3	0	1	2	2	0	0	7 (53.8%)	6 (46.2%)	13 (6.9%)
	41–50	1	4	11	10	6	0	4	5	0	1	1	3	0	1	12 (46.2%)	14 (53.8%)	26 (13.8%)
	51–60	1	0	8	25	3	0	2	19	0	2	3	1	0	3	9 (26.5%)	25 (73.5%)	34 (18.1%)
	61–70	0	8	5	33	2	2	1	26	1	1	1	4	0	0	5 (10.9%)	41 (89.1%)	46 (24.5%)
	71–80	0	6	0	31	0	0	0	21	0	3	0	6	0	1	0 (0%)	37 (100%)	37 (19.7%)
	81–93	0	8	2	18	0	1	1	14	0	0	1	3	0	0	2 (7.1%)	26 (92.9%)	28 (14.9%)
Diabetes	Yes	1	14	15	87	9 *	3	2	66 *	0	5	4	11	0	2	16 (13.7%)	101 (86.3%)	117 (62.2%)
	No	3	12	20	36	8	0	7	22	1	3	4	8	0	3	23 (32.4%)	48 (67.6%)	71 (37.8%)
Hypertension	Yes	1	6 *	11	76 *	4	3	5	61 *	0	3	2	7	0	2	12 (12.8%)	82 (87.2%)	94 (50.0%)
	No	3	20	24	47	13	0	4	27	1	5	6	12	0	3	27 (28.7%)	67 (71.3%)	94 (50.0%)
Cardiac diseases	Yes	1	3	7	37	2	2	4	29 *	0	2	1	3	0	0	8 (16.6%)	40 (83.0%)	48 (25.5%)
	No	3	23	28	86	15	1	5	58 *	1	6	7	16	0	5	31 (22.1%)	109 (77.9%)	140 (74.5%)
Cancer	Yes	0	3	0	3	0	0	0	1	0	0	0	2	0	0	0 (0%)	6 (100%)	6 (3.2%)
	No	4	23	35	120	17	3	9	87	1	8	8	17	0	5	39 (21.4)	143 (78.6%)	182 (96.8%)
Dexamethasone	Yes	3	16	29 *	113 *	14	3	6	82 *	1	6	8	18	0	4	32 (19.9%)	129 (80.1%)	161 (85.6%)
	No	1	10	6 *	10 *	3	0	3	6	0	2	0	1	0	1	7 (25.9%)	20 (74.1%)	27 (15.4%)
Anti-IL-6	Yes	0	0	1	7	1	0	0	6	0	1	0	0	0	0	1 (12.5%)	7 (87.5%)	8 (4.3%)
	No	4	26	34	116	16	3	9	82	1	7	8	19	0	5	38 (21.1%)	142 (78.9%)	180 (95.7%)
Cumulative		4	26	35	123	17	3	9	88	1	8	8	19	0	5	39 (20.7%)	149 (79.3%)	188

^a^ N: Non-fatal, ^b^ F: Fatal. Sex: Use of remdesivir (non-fatal cases: *p* < 0.001, R = 0.598). Remdesivir and favipiravir (non-fatal cases: *p* < 0.048, R = −0.318). Diabetes: Favipiravir (fatal cases: *p* = 0.001, R = 0.381), remdesivir (*p* < 0.001, R = 0.581). Hypertension: favipiravir (fatal cases: *p* < 0.001, R = 0.329). Cardiac diseases: favipiravir (fatal cases: *p* = 0.033, R = 0.166). Dexamethasone: use of antivirals (fatal cases: *p* < 0.007, R = 0.350; non-fatal cases: *p* = 0.007, R = 0.518), favipiravir (fatal cases: *p* < 0.005, R = 0.233).

**Table 3:** Antibiotics used in critical COVID-19 patients with different antiviral regimens.

Variable		No Antivirals (n:30)		Remdesivir (n:20)		Favipiravir (n:97)		Oseltamivir (n:9)		Remdesivir and Favipiravir (n:27)		Favipiravir and Oseltamivir (n:5)		Total Use of Antivirals(n:158)		Total (n:188)
		N *	F **	N	F	N	F	N	F	N	F	N	F	N ^a^	F ^b^	
Antibiotic use	NO	0	0	0	0	0	3	0	0	0	0	0	0	0	3	3 (1.6%)
	YES	4	26	17	3	9	85	1	8	8	19	0	5	35	120	185 (98.4%)
Azithromycin	NO	4	24	16	3	8	83	1	7	6	16	0	3	31	112	171 (90.9%)
	YES	0	2	1	0	1	5	0	1	2	3	0	2	4	11	17 (9%)
Ceftriaxone	NO	4	20	16	3	8	74	1	7	5	11	0	3	30	98	152 (80.8%)
	YES	0	6	1	0	1	14	0	1	3	8	0	2	5	25	36 (19.1%)
Vancomycin	NO	2	18	13	3	6	61	1	6	5	15	0	2	25	87	132 (70.2%)
	YES	2	8	4	0	3	27	0	2	3	4	0	3	10	36	56 (29.7%)
Levofloxacin	NO	3	17	10	3	7	77	1	6	8	16	0	3	26	105	151 (80.3%)
	YES	1	9 ^a^	7	0	2	11	0	2	0	3	0	2	9	18 ^a^	37 (19.6%)
Tienam	NO	4	25	17	3	9	84	1	7	8	19	0	5	35	118	182 (96.8%)
	YES	0	1	0	0	0	4	0	1	0	0	0	0	0	5	6 (3.1%)
Amikacin	NO	4	26	17	3	8	85	1	8	7	17	0	4	33	117	180 (95.7%)
	YES	0	0	0	0	1	3	0	0	1	2	0	1	2	6	8 (4.2%)
Imipenem	NO	4	13	16	3	5	46 ^b^	1	4	7	15	0	3	30	70	117 (94.1%)
	YES	0	13	1	0	4	42 ^b^	0	4	1	4	0	2	5	53	71 (37.7%)
Ciprofloxacin	NO	4	23	16	3	9	82	1	8	8	16	0	5	34	114	175 (93%)
	YES	0	3	1	0	0	6	0	0	0	3	0	0	1	9	13 (6.9%)
Cefipime	NO	4	25	17	3	9	83	1	8	8	19	0	4	35	116	180 (95.7%)
	YES	0	1	0	0	0	5	0	0	0	0	0	1	0	7	8 (4.2%)
Meropenem	NO	4	23	14	2	8	76 ^c^	1	8	6	7 ^d^	0	3	26	96	152 (80.8%)
	YES	0	3	3	1	1	12 ^c^	0	0	2	12 ^d^	0	2	9	27	36 (19.1%)
Tazocin	NO	4	25	16	3	9	87	1	8	8	18	0	5	34	121	184 (97.8%)
	YES	0	1	1	0	0	1	0	0	0	1	0	0	1	2	4 (2.1%)
Moxifloxacin	NO	3	18	11	2	5	48	1	5	0	5	0	1	17	60	98 (52.1%)
	YES	1	8	6	1	4	40	0	3	8 ^e^	14 ^e^	0	4	18	63	90 (47.8%)
Linezolid	NO	2	19	16	3	8	46 ^f^	1	6	6	13	0	4	31	71	123 (65.4%)
	YES	2	7	1	0	1	42 ^f^	0	2	2	6	0	1	4	52	65 (34.5%)
Clindamycin	NO	4	25	17	3	9	85	1	8	8	19	0	5	35	120	184 (97.8%)
	YES	0	1	0	0	0	3	0	0	0	0	0	0	0	3	4 (2.1%)

** F: Fatal cases, * N: No fatal cases. ^a^ Levofloxacin vs. favipiravir in fatal cases (*p* < 0.016, R = −0.175). ^b^ Imipenem vs. favipiravir in fatal cases (*p* = 0.001, R = 0.518). ^c^ Meropenem vs. favipiravir in fatal cases (*p* < 0.016, R = −0.196). ^d^ Meropenem vs. favipiravir and remdesivir in fatal cases (*p* < 0.001, R = 0.423). ^e^ Moxifloxacin vs. remdesivir and favipiravir (fatal cases: *p* < 0.003, R = 0.238 and non-fatal cases: *p* < 0.001, R = 0.521). ^f^ Linezolid vs. favipiravir in fatal cases (*p* < 0.015, R = 0.200).

**Table 4:** Multivariate test of different variables and their effects on the survival rate in patients with severe forms of COVID-19.

Variables	Type III Sum of Squares	df	Mean Square	F	Sig.
Age	107.580	1	107.580	53.409	0.001
Sex	0.355	1	0.355	1.525	0.218
Diabetes	1.810	1	1.810	7.989	0.005
Hypertension	1.922	1	1.922	7.931	0.005
Cardiac diseases	0.124	1	0.124	0.647	0.422
Cancer	0.050	1	0.050	1.619	0.205
Antiviral	0.048	1	0.048	0.358	0.550
Remdesivir	5.343	1	5.343	79.318	0.001
Favipiravir	4.002	1	4.002	17.332	0.001
Tamiflu	0.024	1	0.024	0.529	0.468
Favipiravir and Tamiflu	0.557	1	0.557	1.340	0.249
Remdesivir and favipiravir	4.655	1	4.655	1.510	0.221
Antibiotics	0.013	1	0.013	0.793	0.374
Azithromycin	0.007	1	0.007	0.087	0.768
Ceftriaxone	0.197	1	0.197	1.268	0.262
Vancomycin	0.005	1	0.005	0.022	0.881
Levofloxacin	0.175	1	0.175	1.101	0.296
Tienam	0.050	1	0.050	1.619	0.205
Amikacin	0.004	1	0.004	0.091	0.763
Imipenem	3.062	1	3.062	13.850	0.001
Ciprofloxacin	0.093	1	0.093	1.443	0.231
Cefipime	0.089	1	0.089	2.189	0.141
Meropenem	0.070	1	0.070	0.447	0.505
Tazocin	0.001	1	0.001	0.045	0.833
Moxifloxacin	0.004	1	0.004	0.014	0.906
Linezolid	1.812	1	1.812	8.278	0.004
Clindamycin	0.022	1	0.022	1.064	0.304
Dexamethasone	0.288	1	0.288	2.211	0.139
Anti-IL6	0.024	1	0.024	0.529	0.468

## Data Availability

All data are provided in the manuscript; however, any additional inquiries could be requested from the corresponding author.
